# Bone Health in Patients with Multiple Sclerosis

**DOI:** 10.4061/2011/596294

**Published:** 2011-03-30

**Authors:** Vit Zikan

**Affiliations:** Department of Internal Medicine 3, Faculty of Medicine 1, Charles University, 128 00 Prague, Czech Republic

## Abstract

Multiple sclerosis (MS) is a gait disorder characterized by acute episodes of neurological defects leading to progressive disability. Patients with MS have multiple risk factors for osteoporotic fractures, such as progressive immobilization, long-term glucocorticoids (GCs) treatment or vitamin D deficiency. The duration of motor disability appears to be a major contributor to the reduction of bone strength. The long term immobilization causes a marked imbalance between bone formation and resorption with depressed bone formation and a marked disruption of mechanosensory network of tightly connected osteocytes due to increase of osteocyte apoptosis. Patients with higher level of disability have also higher risk of falls that combined with a bone loss increases the frequency of bone fractures. There are currently no recommendations how to best prevent and treat osteoporosis in patients with MS. However, devastating effect of immobilization on the skeleton in patients with MS underscores the importance of adequate mechanical stimuli for maintaining the bone structure and its mechanical competence. The physical as well as pharmacological interventions which can counteract the bone remodeling imbalance, particularly osteocyte apoptosis, will be promising for prevention and treatment of osteoporosis in patients with MS.

## 1. Introduction

Osteoporosis is a condition of impaired bone strength which leads to increased risk of fracture [1]. The enhanced bone fragility reflects the integration of the amount of bone (bone mass) and bone quality. Bone quality depends on its macro- and microarchitecture and on the intrinsic properties of the materials that comprise it (e.g., matrix mineralization, microdamage accumulation, or collagen quality) [2]. Bone is continually adapting to changes in its mechanical and hormonal environment via the process of bone remodeling. Bone remodeling maintains bone structure and its mechanical competence by removing damaged bone and replacing it with new bone and thus restores bone's material composition, micro-, and macroarchitecture. This process depends on the normal production, work and lifespan of osteoclasts, osteoblasts, and osteocytes. Thus, diseases and drugs that have an impact on bone cells and bone remodeling will influence bone's structure and its resistance to fracture [3]. Multiple sclerosis (MS) is a chronic progressive disease affecting the myelin sheath covering of nerve fibers in the brain and spinal cord, leading to functional impairments such as visual impairment, abnormal walking mechanics, poor balance, muscle weakness, fatigue, and progressive immobilization [4]. The resultant functional impairments lead to frequently falls [5]. The disease affects mainly young adults (20 to 40 years) and its incidence is more frequent in women (approximately 2 : 1) [6]. Its prevalence ranges from 2 to 150 patients at 100 000 [7]. Impaired mobility or lack of weight-bearing physical activity reduced mechanical stress on bone, which causes a marked imbalance in bone remodeling with a disruption of osteocytes network [8]. Management of MS requires long-term disease-modifying therapy, such as glucocorticoids (GCs) with a further negative effect on bone remodeling and bone strength. Secondary osteoporosis may develop and low-trauma fractures occurring in patients with MS more frequently than in healthy controls [9–15]. Fractures and their sequelae can have important personal as well as (economic) implications for society. Therefore, the attention on the issue of bone health among patients with MS is warranted. This paper examines the underlying pathogenic mechanisms of osteoporosis in patients with MS as well as its management. Understanding the causes associated with decreased bone strength in patients with MS will help in the optimal therapeutic intervention. 

## 2. Prevalence of Osteoporosis in Patients with Multiple Sclerosis

The analysis of a registry of 9029 patients with MS in the USA found that 27.2% responders reported low bone mass, and more than 15% of responders reported a history of fracture [9]. Most studies in patients with MS evaluated BMD in comparison with the control group of healthy subjects and showed significantly lower BMD in patients with MS than in controls [11–16]. Several of these studies were shown that vertebral BMD is affected to a lesser degree than femoral BMD [11, 12, 16]. The low BMD in MS patients involve both sexes [15]. Interestingly, one study in men with MS reported low BMD (osteoporosis) in 37.5% (15 out of 40) and 21% (8 out of 38 patients) had vertebral, rib, or extremities fractures [15]. Patients with progressive forms of MS showed a more severe loss of BMD than those with relapsing-remitting MS [12]. Fracture incidence in patients with MS evaluated only few studies. Cosman group found fracture rates of 22% in patients with MS compared with 2% in controls [11]. It remains unexplained whether all patients with MS are more susceptible to osteoporosis and fractures; for example, there is evidence that patients with a low expanded disability status scale (EDSS) score did not show any significant difference in BMD with comparison with healthy control subjects [17, 18]. Therefore, further elucidation is needed to qualify which risk factors are most responsible for a bone loss in patients with MS. 

## 3. Pathogenic Mechanisms

Bone remodeling is under way throughout life and maintains bone strength by removing damaged bone and replacing it with new bone and thus restores bone's micro- and macroarchitecture. This process depends on the normal production, work, and lifespan of osteoclasts, osteoblasts, and osteocytes [19]. Chronic diseases, such as MS, may significantly disturb the process of bone modeling and remodeling with resulting bone loss, deterioration of bone's quality and increased frequency of fractures [20, 21]. Secondary osteoporosis and low-trauma fractures occur in patients with MS more frequently than in healthy controls [9, 11]. The underlying pathogenic mechanisms of the osteoporosis in patients with MS are probably based on the progressive immobilization, long-term GCs treatment, vitamin D deficiency, skeletal muscle atrophy and possibly on the presence of various cytokines involved in the pathogenesis of MS [10]. In addition, chronic use of other drugs, such as antidepressants may contribute to the development of osteoporosis and fractures [22]. The functional impairments also leads to an increased risk of falling that, combined with bone loss and impaired quality of bone mass, can increase the frequency of bone fracture in individuals with MS [11]. 

### 3.1. Disability

Mechanical loading is an important factor controlling bone mass. Increased bone loss in immobilized subjects is well-recognized complication in patients after spinal cord injury with tetraplegia [23, 24], in bedridden patients, or in astronauts [25], whereas localized bone loss is well documented in patients with regional disuse, for example, after fracture itself. Immobilization causes an overall progressive bone loss at a similar rate to osteoporosis caused by estrogen deficiency, but at the same amount of induced bone loss, disuse led to more deteriorated bone structure and mechanical properties than estrogen deficiency [26]. The available studies showed that cortical thinning and substantial decline of trabecular bone density account for increased bone fragility [27–29]. The duration and degree of motor disability appears to be a major contributor to the pathogenesis of secondary osteoporosis in patients with MS. The degree of disability measured by the Kurtzke EDSS score significantly correlated with BMD in patients with MS [11]. Specifically, site-specific effects of motor disability were documented in MS patients, and EDSS correlated mainly with BMD in the hip but not in the lumbar spine [14]. In wheelchair-bound patients, an atrophy of hip muscles affects proximal femur, while BMD of lumbar spine is not decreased because of its adequate mechanical stimulation by the trunk and back muscles in the upright position. Similarly, patients with spinal cord injury lose BMD mainly at femoral sites [30]. Also, hemiplegic patients showed a significant loss of BMD in both trabecular and cortical bone at the forearm and at the neck and great trochanter on the paretic hip [31]. Higher total body bone mineral content was documented in ambulatory patients (EDSS score ≤ 6.5) compared with nonambulatory patients (EDSS score ≥ 7.0) [12, 13]. Also, higher prevalence of osteoporosis was found in nonambulatory patients [32]. In male patients, a positive correlation has been observed between BMD and both EDSS score (correlation with femoral and also vertebral BMD) and BMI (correlation with femoral BMD only). There was shown that also EDSS score and BMI two years prior to the study could be used as future indicators of low BMD [15].

A reduced mechanical stress on bone causes a marked imbalance in bone remodeling with a transient increase in bone resorption (which occurs initially) and a decrease in bone formation (which is sustained for a longer duration) [25, 33, 34] (Table 1). The mechanism causing this decrease in bone formation probably lies in the reduction of mechanical stress during immobilization which results in a marked disruption of osteocytes network due to increase of osteocyte apoptosis. Osteocytes represent 95% of all bone cells and form a mechanosensory system which is based on a three-dimensional network of tightly interconnected osteocytes entombed in mineralized bone matrix [35]. Disruption of this system affects probably several aspects of bone homeostatic system, such as mechanosensitivity, mechanotransduction, and basic multicellular units responsible for bone remodeling [36]. The immobilization-induced osteocyte apoptosis is followed by osteoclastogenesis and increased bone resorption [37]. While molecular mechanisms of disuse osteoporosis are not well understood, recent evidence found that mechanical unloading caused upregulation of *Sost* gene in osteocytes and increased levels of sclerostin (product of *Sost* gene) [38]. Sclerostin is responsible for the inhibition of Wnt/beta-catenin signaling in vivo and for the suppressed viability of osteoblasts and osteocytes. Interestingly, sclerostin-deficient mice (*Sost *−/−) were resistant to mechanical unloading-induced bone loss [38]. Importantly, the administration of sclerostin neutralizing antibody in experimental model of immobilization resulted in a dramatic increase in bone formation and a decrease in bone resorption that led to increased trabecular and cortical bone mass [39]. Osteocytes are also necessary for targeted bone remodeling to avoid microdamage accumulation, which could lead to whole-bone failure. Recently, Waldorff et al. showed that osteocyte apoptosis may be insufficient for repair of microdamage without the stimulation provided through physiologic loading [40]. MS affects a wide range of neurological function and most of patients with MS have abnormal muscle strength, impaired balance, and gait control which leads to frequent falls [5, 41] that combined with a bone loss increase the frequency of bone fractures. Imbalance is also often the initial symptom of MS. The pathogenesis is not completely understood yet. It was demonstrated that changes in postural control in most patients with MS are probably the result of slowed afferent proprioceptive conduction in the spinal cord [5]. Disuse, inflammatory changes, as well as GCs treatment or vitamin D deficiency, may also contribute to weakness and loss of muscle strength and thus to frequent falling. 

### 3.2. Glucocorticoids (GCs)

GCs are frequently used to control MS relapses. Oral GCs treatment in patients with MS may increase the risk of osteoporosis. Epidemiological studies showed that fracture risk is increased rapidly after starting oral GCs treatment and is related to the dose and duration of GCs exposure [42]. Doses as low as 2.5–5 mg of prednisolone equivalents per day can be associated with a 2.5-fold increase in vertebral fractures, and the risk is greater with higher doses used for prolonged periods [43]. Bone loss due to GCs treatment is steep during the first 12 months and more gradual but continuous in subsequent years. However, the fracture risk returns towards baseline levels after discontinuation of oral GCs treatment [44]. The mechanism of osteoporosis in patients on GC treatment is complex [45] (Table 2). However, the contribution of other risk factors, such as vitamin D insufficiency and physical disability confounds the assessment of GCs effects on bone in patients with MS. 

Repeated pulses of high-dose methyprednisolone in MS patients did not result in a subsequent decrease in BMD [18]; however, the risk of osteoporotic fractures remains slightly increased in patients undergoing cyclic GCs treatment at high doses [47]. High-dose, short-term intravenous GC regimens cause an immediate and persistent decrease in bone formation and a rapid and transient increase of bone resorption [48]. In fact, GCs may increase proresorptive IL-6 signaling as well as increase the expression of receptor activator of NF-*κ*B ligand (RANKL) and decrease the expression of its soluble decoy receptor, osteoprotegerin (OPG), in stromal and osteoblastic cells [49]. Moreover, GCs may directly decrease apoptosis of mature osteoclasts [50]. However, discontinuation of such regimens is followed by a high bone turnover phase [48]. In physically active patients with MS treated with low-dose steroids, the bone turnover markers were not different from controls [51]. Addressing the question of whether duration of low-dose GCs use in combination with other immunomodulators in patients with MS increases risk of osteoporosis requires further prospective study by taking into account other risk factors, particularly the level of disability. 

### 3.3. The Effect of Other Immunomodulatory Drugs

Although no harm effect of low-dose methotrexate was observed in patients with MS, several case reports have described associations between pathological nonvertebral fractures and low-dose methotrexate (MTX) in rheumatoid arthritis (RA) patients [52]. In addition, methotrexate osteopathy, characterized by pain, osteoporosis, and microfractures, has been very rarely observed in patients with low-dose MTX treatment [53]. Other immune-modifying drugs, such as interferon-beta or azathioprine, which are used in conjunction with GCs have not been shown to promote bone loss experimentally or clinically. On the contrary, interferon-beta may have favorable effect on bone metabolism in patients with MS [54], probably due to the inhibitory effect of interferon-beta on osteoclasts development [55]. Experimentally, also treatment with the S1P(1) agonist FTY720, a new and promising drug for the treatment of MS, relieved ovariectomy-induced osteoporosis in mice by reducing the number of mature osteoclasts attached to the bone surface [56]. However, further investigation with regard to their effects on bone health is needed. 

### 3.4. Vitamin D Insufficiency

The role of vitamin D in bone homeostasis is well understood, and the use of vitamin D to prevent and treat osteoporosis was recently reviewed [57]. There is also evidence from both observational studies and clinical trials that hypovitaminosis D are predisposing conditions for various common chronic diseases. In addition to skeletal disorders, vitamin D deficiency is associated with increase the risk of malignancies, particularly of colon, breast, and prostate gland cancer, of chronic inflammatory and autoimmune diseases (e.g., insulin-dependent diabetes mellitus, inflammatory bowel disease, or multiple sclerosis), as well as of metabolic disorders (metabolic syndrome and hypertension) [58]. Vitamin D intake, decreasing latitude, increased sun exposure, and high serum vitamin D levels have all been shown to be associated with a decreased risk of MS [59]. Patients with MS have more often vitamin D deficiency due to its low intake as well as limited sunlight exposure [12]. Mean 25-hydroxyvitamin D_3_ (25OHD) levels in patients with MS are more often lower (below the level of 20 ng/mL) than in age-matched controls [11, 14]. There was no significant correlation between 25(OH)D and BMD in patients with MS [11, 14]. Thus, while patients with MS are susceptible to low 25OHD levels, the evidence implicating linking levels to reduced BMD and osteoporosis in patients with MS is unclear. Only a few studies have investigated this link [11, 12, 14]. A low vitamin D state, from inadequate diet intake and decreased exposure to sunlight, contributes to malabsorption of calcium and vitamin D insufficiency in MS patients. Secondary hyperparathyroidism may develop, which can contribute to bone remodeling imbalance and bone loss in patients with MS. Moreover, patients with MS treated with GCs will be at greater risk for an imbalance between bone formation and bone resorption and, therefore, more susceptible to development of osteoporosis due to vitamin D insufficiency/deficiency. GCs treatment is associated with reduced calcium absorption from the gastrointestinal tract by opposing vitamin D action. Furthermore, renal tubular calcium reabsorption is also inhibited by GCs. In addition, GCs may affect PTH secretory dynamic, with a decrease in the tonic release of PTH and an increase in pulsatile burst of the hormone [46]. 

### 3.5. The Chronic Inflammatory Process of Multiple Sclerosis

MS is an inflammatory disease of the central nervous system (CNS) with a prominent role of immune cells and cytokines in degradation of the myelin sheaths [60]. Recent evidence has indicated that a number of additional cell types, such as T cells, play a key role in bone loss [61]. In inflammatory or autoimmune disease states, activated T-cells produce receptor activator of nuclear factor kappaB ligand (RANKL) and proinflammatory cytokines, such as TNF-*α*, IL-1, or IL-11, all of which can induce RANKL expression in osteoblasts and bone marrow stromal cells. The systemic or local activation of T-cells may, therefore, trigger bone loss via the expression of RANKL [61]. Osteoprotegerin (OPG), a protein member of the tumor necrosis factor (TNF) receptor family and its ligand RANKL were identified as a key cytokines that regulate osteoclastogenesis [61]. Significantly, higher levels of RANKL and OPG were found in the patients with MS with low mean EDSS as compared to the age-matched controls [62]. Among other cytokines, osteopontin (OPN) has been studied in the shared pathogenesis of MS and osteoporosis. OPN is a member of the SIBLING (small integrin-binding ligand N-binding glycoprotein) family of noncollagenous matricellular proteins [63]. OPN was identified as the most abundantly expressed cytokine in MS lesions, and OPN levels were found to be increased in cerebrospinal fluid of MS patients [64, 65] and in the plasma in patients with relapsing-remitting MS [66]. However, other studies found that OPN circulating levels are low in patients with MS [67]. It seems likely that further future studies experiments will uncover the role of OPN and additional molecules mediating bone loss in inflammatory diseases, such as MS. 

### 3.6. Use of Antiepileptic and Antidepressant Drugs

Antiepileptic drug treatment can lead to osteoporosis [68, 69]. Meta-analyses have revealed that barbiturate, antidepressant, antipsychotic, and benzodiazepine treatment increases patient's risk of osteoporosis [70]. More recently, current use of antidepressant drugs with a high affinity for the 5-hydroxytryptamine reuptake transporter (5-HTT) was associated with a higher risk of osteoporotic fractures compared to use of antidepressants with a medium or low affinity [71]. BMD was lower among those reporting current selective serotonin reuptake inhibitors (SSRI) use but not among users of other antidepressants [72, 73]. In vivo studies have found that 5-HT could alter bone architecture and could reduce bone mass and density [74]. The 5-HTT has been located in osteoclasts, osteoblasts, and osteocytes, and the the inhibition of 5-HTT using a SSRI (fluoxetine hydrochloride) had antianabolic skeletal effects in rats [74]. Further research is needed to confirm this finding in light of widespread SSRI use and potentially important clinical implications. 

## 4. Diagnosis and Management of Osteoporosis in Patients with MS

Despite the fact that patients with MS can develop osteoporosis and fractures more often than their age-matched healthy controls, many patients with MS are not evaluated for their bone status, and there are no clinical guidelines for prevention and treatment of osteoporosis in patients with MS. Patients with MS are also at a higher risk of falls that can increase the frequency of bone fracture combined with bone loss and impaired bone's quality. 

Clinical evaluation in all patients with MS should include the assessment of the clinical risk factors for osteoporosis and fractures, such as the hereditary disposition of osteoporosis, previous low trauma fractures, and smoking or alcohol habits. The specific risk factors of the osteoporosis in patients with MS are the level of disability (specifically motor disability) and possibly a long-term GCs treatment, vitamin D deficiency, skeletal muscle atrophy, and increased risk of falling. The examination of the motor function using the EDSS score could provide a useful indicator for further evaluation. Cutoff EDSS 6 represents reasonable end of motor performance of the patient; 6.5 means only several meters with bilateral support, and 7 is only the ability of transfer to wheelchair from the bed. The EDSS scores of 6 or greater has been found to correlate well with decreased BMD [12, 15], and BMD should be routinely measured in these patients. On the other hand, patients with a good physical activity and low EDSS score (<5) may have normal BMD [14] as well as markers of bone turnover [51]. BMD measurement should be also performed in all patients who are receiving 5 mg of prednisone equivalents daily for more than 3 months.

BMD testing using dual-energy X-ray absorptiometry (DXA) should be conducted at the lumbar spine and hip. This measure provides an assessment of fracture risk prior to the occurrence of a fragility fracture as well as monitors the course of the disease and response to therapy. No consensus exists as to how frequently patients at risk osteoporosis should have followup scans. However, BMD should be remeasured after 1 or 2 years to ascertain that it is stable or to identify the patient with ongoing bone loss, especially in patients treated with long-term GCs treatment. In the presence of clinical risk factors, fracture risk may be increased independently from BMD. Therefore, combination of BMD with clinical risk factors is recommended to identify a risk patient and to target pharmacologic therapy. In postmenopausal women and men (between 40 and 90), the assessment of individualized 10-year absolute fracture risk (FRAX, fracture prediction algorithm) is recommended [75].

The identification of previous low-trauma fractures, especially vertebral fractures is important for the decision-making process as a previous vertebral fracture is a particularly strong risk factor. Importantly, vertebral fractures may occur in 30–50% of patients receiving chronic GCs therapy [76] and up to 50% of vertebral fractures are asymptomatic and, therefore, do not come to the attention of physicians. Spinal X-rays should be performed in those with localized back pain or a loss of more than 3 cm in height in order to detect prevalent vertebral fractures. Alternatively, the vertebral fracture assessment tool of the bone densitometer, which is associated with low radiation, may be useful screening test for vertebral fractures assessment.

 Laboratory tests are indicated to exclude other secondary causes of osteoporosis, such as vitamin D deficiency, renal insufficiency, malabsorption, and hypogonadism. Useful biochemical tests include routine standard tests to exclude renal or hepatic impairment, blood count, serum calcium, 24-hour urinary calcium, 25-hydroxyvitamin D_3_ (to exclude vitamin D deficiency), and gonadal hormones (to exclude hypogonadism). 

## 5. Treatment Options

### 5.1. Nonpharmacological Considerations

Prevention is more effective than treatment of established osteoporosis. For all patients, nonpharmacological therapies should be considered for prevention of skeletal fragility, including adequate weight-bearing exercise, nutrition (protein, calcium, vitamin D), and lifestyle modifications. As reviewed above, disability is the most often cause of bone loss in patients with MS, and mechanical loading and exercise interventions can prevent osteocyte apoptosis and bone loss [77, 78]. Exercises have beneficial effects on strength, physical endurance, mobility-related activities (transfer, balance, and walking), and on mood, without any evidence of detrimental effects [79]; however, there was no evidence that any particular exercise programs were more effective in improving or maintaining function. Whole-body vibration is a new approach to improve neuromuscular functions and bone strength, but there is limited evidence that whole body vibration provides any additional improvements [80]. Further experimental studies are necessary to identify optimal physical activities for the prevention of osteocyte apoptosis and bone loss. Recurrent falls may be an important risk factor for fracture in disabled patients with MS. In patients with MS, falls are related to the level of disability [81], and possibly other factors may contribute to muscle weakness and imbalance, such as vitamin D deficiency or GCs treatment. 

### 5.2. Calcium and Vitamin D

Calcium and vitamin D supplementation has been routinely provided in most clinical trials of bone protective therapy for both primary and secondary osteoporosis, for example, in glucocorticoid-induced osteoporosis (GIO). The effect of calcium and vitamin D supplementation is maximized in patients whom baseline intake is low. As patients with MS are at a higher risk of calcium and vitamin D deficiency should have their calcium and vitamin D status checked and intake must be individualized. Those with a personal or family history of nephrolithiasis must be screened with 24-h urinary calcium. In immobilized patients, an increase in serum calcium is provoked by bed rest alone and additional calcium intake would not be helpful and might be harmful and provoke an increased risk of kidney stone formation. However, calcium and vitamin D should be used as an adjunct treatment, because a low calcium intake may exacerbate calcium loss during low mechanical loading [82]. In general, the amount of vitamin D supplementation should aim at achieving serum 25OHD levels above 50 nmol/l in >95% of adults without causing vitamin D toxicity. A daily dose of 800–1000 IU of vitamin D3 should be able to obtain this minimal 25OHD target. Due to vitamin D resistance in patients receiving GCs, those patients may require amounts of 1000–2000 IU of vitamin D3 daily [83]. Measurement of serum 25OH vitamin D is recommended, especially in GCs-treated patients. Although some evidence suggests that daily supplemental intake of 2000–4000 IU colecalciferol is required to obtain at least 75 nmol/l 25OHD, which may be optimum for many health outcomes [84], prospective trials showing that higher 25OHD levels (>75–80 nmol/l) are conveying additional benefits without new risk are needed. 

### 5.3. Pharmacological Interventions

The ultimate goal of all pharmacological interventions is prevention of fractures. Although a number of drugs have been evaluated for the prevention and treatment of postmenopausal osteoporosis and GIO, the evidence of their efficacy in patients with MS, especially in premenopausal women and younger men is less strong. As osteoporosis in MS patients have multiple pathogenesis, medical interventions used in women with postmenopausal osteoporosis may not be similarly efficient. Patients requiring long-term GCs treatment and those being immobilized may require pharmacological therapy to prevent excessive bone loss and fractures. Options for treatment include antiresorptive drugs, such as estrogen, or aminobisphosphonates, or anabolic agents such as teriparatide.

*Aminobisphosphonates* (BPs). Although the use of BPs may be appropriate, the etiology of osteoporosis in patients with MS is fundamentally different from the osteoporosis commonly found in the postmenopausal women for whom these drugs were originally developed. As immobilization in patients with MS can cause substantial bone loss and increase in the risk of fractures [20], BPs may be option for treatment for those patients. Although BPs have not been systematically evaluated in the therapy of these conditions, some studies support the potential benefit of BPs in the management of bone loss associated with immobilization [24, 85, 86]. In immobilized patients, BPs is known to reduce immobilization-induced hypercalcaemia by inhibiting bone resorption of calcium. An immobilization-related elevated serum calcium level may inhibit parathyroid hormone (PTH) secretion, and hence renal 1, 25(OH)_2_D_3_ production, in disabled long-standing MS patients. If oral therapy of BPs cannot be tolerated or excluded due to gastroesophageal disease, intravenous route of administration of ibandronate or zolendronate may be applied. However, acute phase reaction with fever, particularly after the first application of BP, may occur. BPs (alendronate, risendronate, or zolendronate) were also approved for the treatment of GIO. These drugs were shown to improve BMD, whereas the data on fractures were scanty in GIO, particularly in premenopausal or younger men. The mechanism by which BPs reduce the adverse skeletal effects of GCs have not been elucidated. The disadvantage of long-term BPs treatment is that it may lead to a reduction in bone turnover to a level inadequate to support normal bone remodeling. Although experimental data showed that BPs also prevents osteocyte apoptosis, there is also experimental evidence of increased accumulation of microdamage with long-term BPs therapy [87]. Also, as BPs accumulate in the skeleton (with a long-term residual time), they cross the placenta, accumulate in fetal skeleton, and cause toxic effects in pregnant rats. Therefore, BPs should be used with caution in women who may become pregnant. 

### 5.4. Anabolic Drugs

Drugs, such as BPs, that suppress bone resorption have been proposed as interventions for prevention of GIO as well as disuse osteoporosis. The disadvantage of this approach is that it may lead to a reduction in bone turnover to a level inadequate to support normal bone remodeling. An alternative approach is to maintain a normal level of bone formation using a bone anabolic agent such as PTH. The human recombinant N-terminal parathyroid hormone (PTH 1–34 or teriparatide) is a potent osteoanabolic agent, which decreases osteoblast and osteocyte apoptosis and increases bone formation and bone strength. Because of GCs-induced decrease in the number of osteoblasts and rate of bone remodeling, anabolic, and antiapoptotic treatment with teriparatide may directly counteracts the key pathogenetic mechanisms of GCs excess on bone, thus, it may be a more effective treatment than BPs [88]. The same rationale applies to immobilization-induced osteoporosis, as progressive immobilization as well as long-term GCs exposure results in osteocyte apoptosis and reduced bone formation [89]. 

### 5.5. Future Options

As sclerostin augments osteocyte apoptosis, the antibody-mediated blockade of sclerostin represents a promising new therapeutic approach for the anabolic treatment of immobilization-induced osteoporosis and probably also for GCs-induced osteoporosis. Indeed, more recently, experimental data showed that administration of sclerostin neutralizing antibody in rat model of right hindlimb immobilization resulted in a dramatic increase in bone formation and a decrease in bone resorption that led to increased trabecular and cortical bone mass [39]. 

## 6. Summary

We have described a spectrum of pathogenetic factors which may contribute to the development of osteoporosis and low-trauma fractures in patients with MS. Whilst there is evidence to support an important role for many of the risk factors, the most significant etiology of bone loss in patients with MS seems to be the level of motor disability and reduced bone load within individual patients. Other risk factors, such as long-term GCs treatment, hypovitaminosis D, or inflammation, may also play an important part in subset of patients with MS; however, further examinations in prospective studies are required. With regard to diagnostic as well as therapeutic interventions, there are currently no specific recommendations in patients with MS; however, identification and treatment of underlying cause should be the goal of therapeutic management. Optimally, the patients in a higher risk of osteoporosis should be early identified and preventively promptly treated to avoid the bone loss and fractures. Because the long-term disability and long-term GCs are probably two most significant etiologic risk factors for osteoporosis development in the majority of the patients with MS, the interventions which can counteract the osteocyte apoptosis as well as loss of muscle mass and muscle weakness will be promising. 

## Figures and Tables

**Table 1:** Changes in bone cells metabolism and in bone mass/structure in patients with long-term immobilization.

	Increased	Decreased
Osteocytes	Apoptosis	Metabolism and function
		Repair of microdamage
Osteoblasts	Apoptosis	Activity
		Synthesis of type I collagen
Osteoclasts	Activity	Apoptosis
Bone homeostasis	Remodeling rate	
	Bone resorption	Bone formation
		Bone mineral density*
		Cortical thickness*
		Cortical density*
		Trabecular density*

*The anatomic location and function of the bone in the skeleton account for the magnitude of skeletal response to immobilization.

**Table 2:** The mechanisms of bone loss during long-term GCs treatment.

	Inhibition	Stimulation
*Bone cells direct effects*		
Bone marrow/stromal cells	differentiation into	differentiation into
	osteoblasts	adipocytes
Osteoblasts	differentiation, activity	—
	synthesis of type I collagen	apoptosis
Osteocytes	metabolism and function	apoptosis
Osteoclasts	apoptosis	stimulation

*Indirect effects*		
Gut	Ca^2+^ absorption	—
Renal tubule	Ca^2+^ reabsorption	—
Parathyroid-PTH	Tonic secretory rate*	Pulse secretory
rate*		
		Fractional pulsatile
secretion*		
Pituitary	Growth hormone/IGF-1	—
	FSH, LH	—
Testes, ovaries	Testosterone, estradiol	—

*Data from Bonadonna et al. [46]; abbreviations: FSH: follicle stimulating hormone; LH: luteinizing hormone; IGF-1: insulin like growth factor 1.
